# Dr. Pearson and the I. H. A.

**Published:** 1884-01

**Authors:** Ad. Lippe

**Affiliations:** Philadelphia


### DR. PEARSON AND THE I. H. A.

Dr. C. Pearson, at the instigation of prominent members of the I. H. A., and as executive officer of the Association, has arraigned the undersigned before the tribunal of the medical profession, in THE HOMŒOPATHIC PHYSICIAN, Vol. III, p. 361. The arraignment is:

*First*, to have attempted to establish as an "historical fact" that the I. H. A. has, by resolution or otherwise, indorsed the opinions of Dr. Swan.

*Second*, to have set up a man of straw (Samuel Swan?), on which he has trained his artillery for over a year.

*Third*, that the declarations by the I. H. A. contain publicly expressed opinions that it was the original object of the I. H. A. to reform the Institute are false.

*Fourth*, that too much noise has been made over the new heresy.

To the *first and second* points we answer: For over a year the homœopathic profession has been disgraced by an attempt to set up newly discovered laws, viz.: That the morbific product of a disease will cure the disease itself if said product is highly potentized. This assertion has been made by Dr. Samuel Swan in print over and over again. There may be a difference of opinion on that subject. Dr. Pearson may cunningly try to defend this heresy, and appeal to a sentence at the end of the first volume of *Chronic Diseases* by Samuel Hahnemann. For my own part, I take the liberty of denouncing now, as severely as ever before, this heresy. I call the attention of the executive officer of the I. H. A., and of the prominent members of the Association who sustain him, to a simple documentary evidence. *Vide Hahnemann's Organon*, original German, fourth edition, page 67, or the fourth American edition, page 86, or even that mistranslation by C. Wesselhœft, page 59. Hahnemann there fully exposes the fallacy of Lux, who attempted to proclaim and substitute æqualia æqualibus as the true principle in therapeutics, exactly as Swan does now. Two years later he wrote the *Chronic Diseases*. He there sets forth that he has omitted Psorinum from among the antipsorics because it *was not sufficiently proven*. (He did not say because it was not sufficiently potentized.) Hahnemann was very severe in his denunciation of that heresy, and if his words will bring comfort to Drs. Swan, Pearson, and others they must be ready to be comforted by harsh handling. We did say that the I. H. A. *sanctioned* Dr. Swan's heresies, and came to that conclusion because of the silent consent given to the published heresies by a member of the Association; but now we have to thank the executive officer who now unhesitatingly says, "It is not proposed in this connection to defend Dr. Swan; he is amply competent to do that for himself." Dr. Pearson here plainly admits that Dr. Swan's heresy is *defensible*, because it is all right and *indorsed now* by the executive of the I. H. A. We now say the I. H. A. *indorses* every heresy Dr. Swan has seen fit to advertise, and have used as proof of this assertion "documentary evidence." To the third point we answer: At the beginning of the organization of the I. H. A., the question was asked by the presiding officer (Dr. P. P. Wells), "What is the object of this Association?" The accused made then the very first declaration, and was not contradicted by Dr. Pearson. The object, as by him stated, was to attempt to "reform the Institute," and that it was by no means to be inferred that a secession from said Institute was contemplated. We thank Dr. C. Pearson for the information he gives us now—that this was *not* the object. We knew that long ago, and because we were *mistaken* as to the real object of the I. H. A. we "resigned our connection with it."

*Fourth*, that too much noise has been made over the new heresy. To this accusation we plead guilty! The Lux heresy died a natural death over half a century ago, and being resuscitated now by a philosopher in Gotham gives no assurance that it will live any longer than the Lux mania; and really we feel sorry to have wasted so much ammunition without even convincing so astute a healer as is Dr. Pearson that Dr. Swan's discoveries are utterly ridiculous on the face of them. And taking leave of our friend, Dr. Pearson, we have only to say to him that the Lippe Society which he mentions has never even intimated that it is a reformatory body—it is composed of a body of men who believe in the teachings of Hahnemann; who have the moral courage to form a social club; who mind their own business and discuss progressive Homœopathy. We leave eclectics, allopathists, isopathists, specificists, quacks, and faith-curers alone; give them rope enough, we say, and they will hang themselves.

Dr. Gregg strictly adheres to the teachings of Hahnemann, and is true to the cause; Dr. Swan sets aside all of Hahnemann's teachings, and is not true to the cause.

And as Dr. C. Pearson has changed his mind as to the policy of "masterly inactivity" he has so stubbornly followed for years, we may hope that at a future day he may change his mind and abandon his advocacy of isopathy and kindred heresies, and remember that truth and error can never co-exist together, and that you cannot touch pitch without soiling your fingers.

PHILADELPHIA, *Dec. 7th*, 1883. AD. LIPPE.

